# Artificial intelligence and chatbots in general surgery: a survey among surgeons in Germany, Austria and Switzerland

**DOI:** 10.1007/s00464-026-13044-5

**Published:** 2026-07-01

**Authors:** Sebastian Lünse, Eric L. Wisotzky, Lasse Renz-Kiefel, Christoph Paasch, Richard Hunger, René Mantke

**Affiliations:** 1 Department of General and Visceral Surgery, Brandenburg Medical School Theodor Fontane, University Hospital Brandenburg/Havel, Hochstrasse 29, 14770 Brandenburg, Germany; 2 Vision and Imaging Technologies, Fraunhofer Heinrich-Hertz-Institut HHI, Einsteinufer 37, 10587 Berlin, Germany; 3 Department of Computer Science, Humboldt-Universität Zu Berlin, Unter Den Linden 6, 10117 Berlin, Germany; 4 Faculty of Health Science Brandenburg, Brandenburg Medical School Theodor Fontane, University Hospital Brandenburg/Havel, 14770 Brandenburg, Germany

**Keywords:** Artificial intelligence, Large language models (LLMs), Survey, General surgery

## Abstract

**Background:**

The integration of artificial intelligence (AI) and large language models (LLMs) into surgical practice is increasingly being explored, but real-world usage patterns and barriers remain insufficiently characterized. This multinational survey assessed self-reported AI/LLM use, perceived benefits, and requirements for routine implementation among general surgeons in German-speaking countries.

**Methods:**

Between June and September 2025, a 16-item online survey was conducted among general surgeons at university hospitals in Germany, Austria, and Switzerland.

**Results:**

Of 3831 invited surgeons, 323 complete responses were analyzed (response rate 8.7%). Self-reported AI use was frequent: 58.5% reported occasional and 28.2% regular use. The most frequent applications were speech recognition (65.3%) and chatbots (62.8%). Anticipated benefits focused on documentation simplification (94.4%), reduced administrative time (84.2%) as well as burden (83.0%), and improved diagnostic accuracy (70.6%). ChatGPT was the leading chatbot (89.8%), chatbot use was rated helpful (69.6%), and the most common use case was scientific writing (51.4%). Key barriers to routine AI adoption were insufficient integration into existing systems (77.1%), legal/data-protection uncertainty (65.9%), and lack of validated applications (59.1%). The most important requirements were system reliability (76.2%), a clear legal framework (72.1%), improved technical infrastructure (68.4%), and transparency (58.5%). Most respondents expected AI to improve surgical care quality (82.4%) and endorsed structured AI training (85.1%).

**Conclusion:**

The use of AI and LLM-based chatbots was commonly reported among general surgeons in German-speaking countries, primarily for low-threshold, efficiency-oriented tasks such as speech recognition, documentation, scientific writing, and other text-based productivity. Broader integration into surgical workflow will require interoperable implementation, validated clinical-grade applications, legal clarity, and structured education.

**Supplementary Information:**

The online version contains supplementary material available at https://doi.org/10.1007/s00464-026-13044-5.

The integration of artificial intelligence (AI) and, more recently, large language models (LLMs) into general surgery is increasingly being explored across multiple domains of the surgical workflow, including documentation, literature synthesis, perioperative decision support, and image-based applications [1–4]. Within this broad field, LLMs process and generate human language and have enabled natural-language interfaces for clinical and scientific writing, patient communication, education, and research workflows, whereas machine learning (ML)-based predictive models and computer vision (CV) approaches are more commonly applied to structured data analysis and visual interpretation [5–7].

Nevertheless, the real-world adoption of AI in surgery is contingent on numerous factors beyond the performance of the algorithm. Surgeons must contend with data protection, legal liability, model transparency, and cross-institutional validation, while LLM-based documentation tools introduce further concerns regarding factual reliability, bias, authorship, and user training. While other specialties have surveyed this topic [8–11], empirical evidence remains scarce on how general surgeons use AI, which tasks they deem most promising, and which barriers limit routine adoption.

To address this gap, we conducted a multinational survey among surgeons in Germany, Austria, and Switzerland to assess the current and intended use of AI and LLM-based chatbots, perceived benefits within and beyond patient care, and requirements for broader AI adoption.

## Materials and methods

### Survey

The prospective online survey was conducted in accordance with the General Data Protection Regulation (GDPR) using the “SoSci Survey” web application, a commercially available e-survey platform [12]. The results were presented in adherence with the Checklist for Reporting Results of Internet E-Surveys (CHERRIES) [13]. An email invitation was distributed between June 26 and September 26, 2025, to all publicly accessible email addresses of general surgeons at 38 university hospitals in Germany, five university hospitals in Austria, and five university hospitals in Switzerland. The 16-item questionnaire was administered in German and permitted single or multiple response items, depending on the question type (see Supplementary Table 1). The survey was designed as an exploratory instrument for this study, and thus no formal pilot testing or psychometric validation (e.g. reliability or construct validity assessment) was performed. Prior to commencing the survey, all participants were informed that their involvement was entirely voluntary and anonymous. Respondents indicated their position within the typical surgical hierarchy in German-speaking countries, defined as: (a) resident physician—surgical trainee without board certification, (b) consultant—board-certified surgeon working under supervision, (c) senior physician—board-certified surgeon supervising resident physicians and consultants, (d) head physician—senior surgeon leading a surgical department. As this study involved exclusively anonymized data from medical professionals, a waiver was approved by the local ethics committee (E-01–20230705).

### Data analysis

Descriptive statistics were used to summarize survey responses, with categorical variables reported as absolute numbers and percentages. Comparisons between groups defined by level of surgical training (resident physicians, senior physicians, consultants, and head physicians) were performed using the chi-square (*χ*^2^) test for categorical variables. When appropriate, tests for trend across ordered groups were applied to evaluate associations between increasing professional experience and response patterns. All *p*-values are reported two-sided, and a *p*-value < 0.05 was considered statistically significant. Because of the exploratory design of the study, no adjustment for multiple tests was performed. Respondents with incomplete questionnaires were excluded from the analysis. Figures and Supplementary Tables were used to illustrate response distributions and group-wise comparisons. Data analysis was performed with R version 4.4.3 (R Software Foundation, Vienna, Austria).

## Results

### Demographics of the participating surgeons

**Fig. 1:**
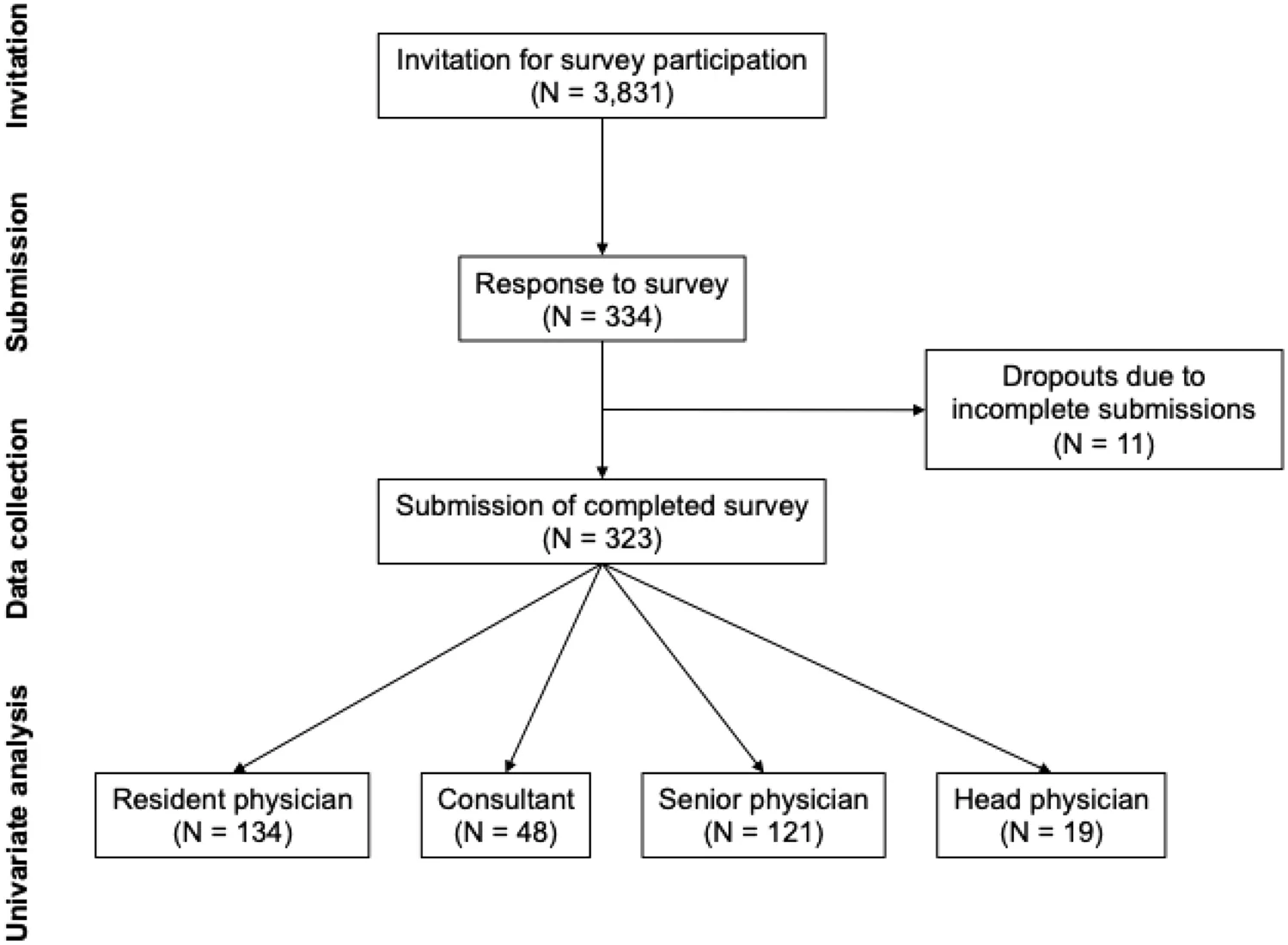
Trial scheme of the survey

**Fig. 2:**
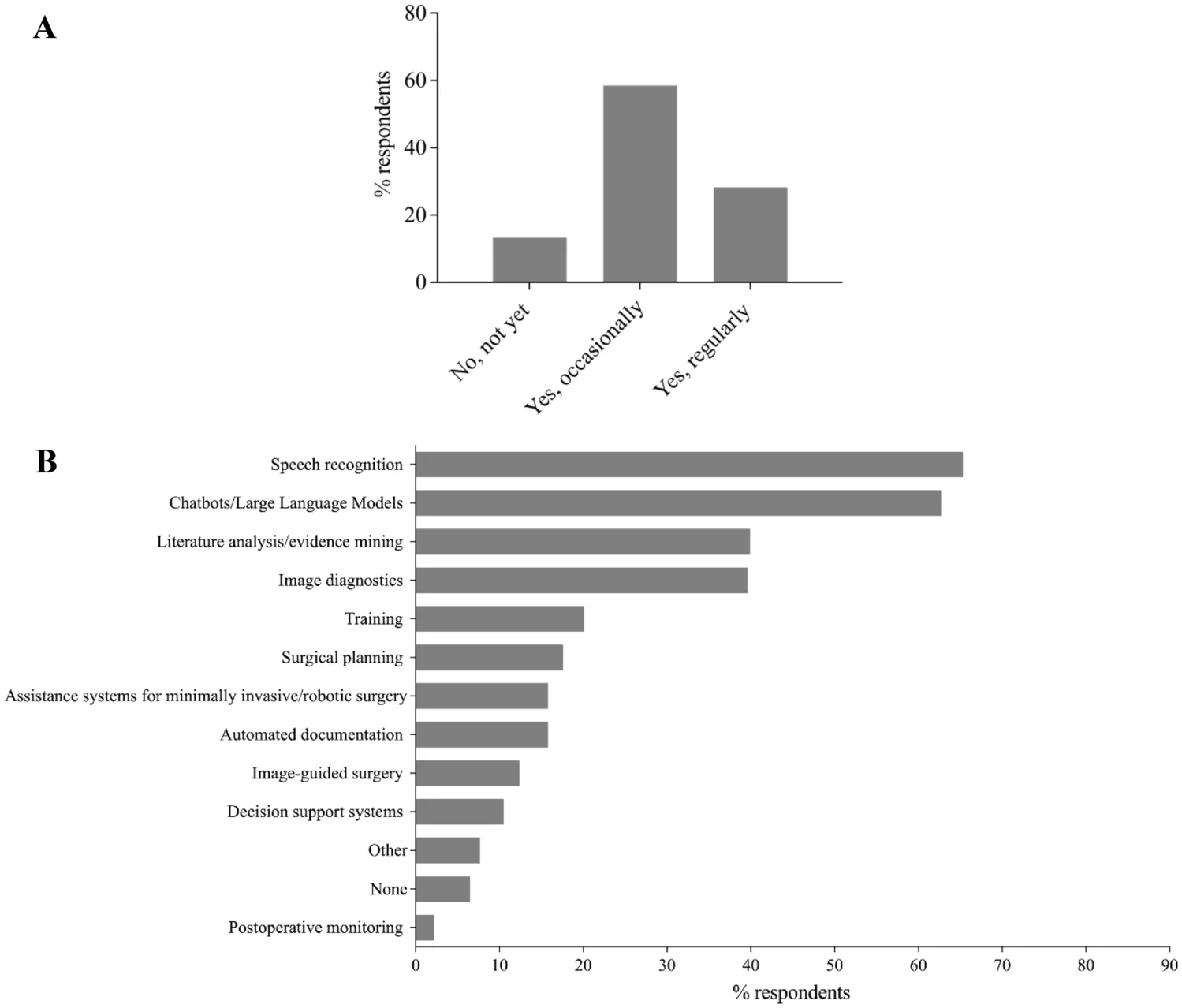
**A** Respondents’ experiences with AI, and **B** areas of previous AI application; multiple selections were possible for **B**

**Fig. 3:**
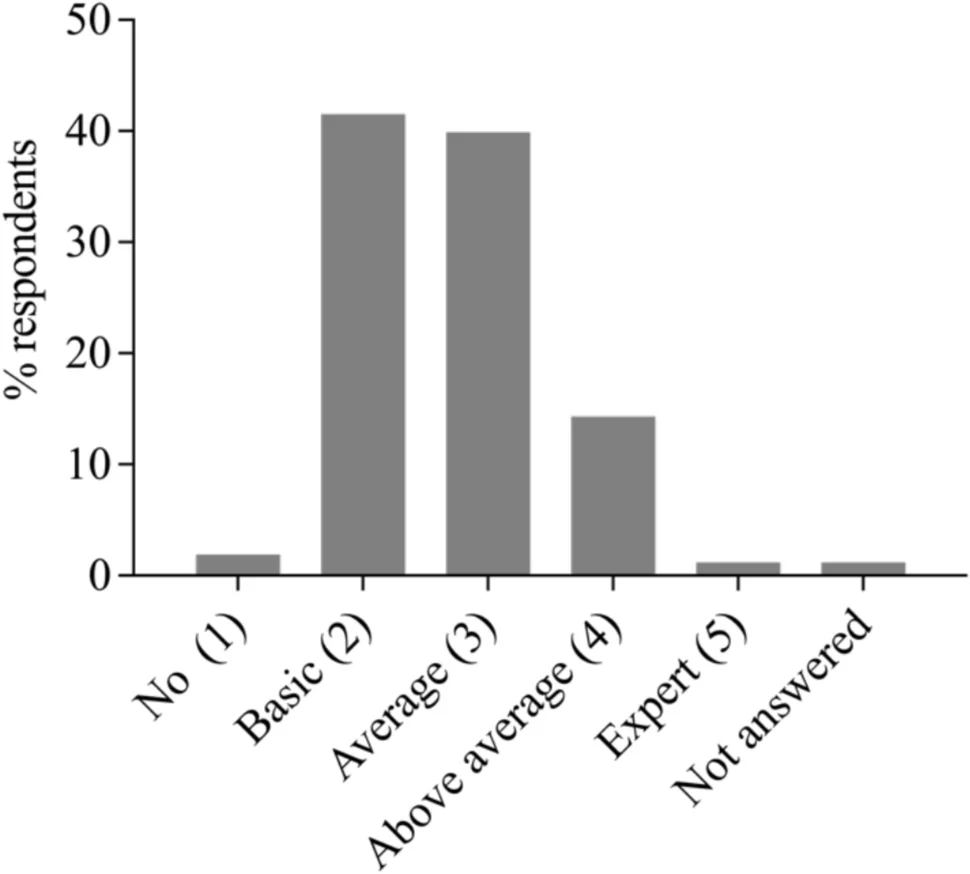
AI knowledge as assessed by the respondents themselves

From the 3831 invited surgeons, 334 responded (8.7%), of whom 323 had complete data and were analyzed (Fig. 1). The majority of respondents were resident physicians (*n* = 134; 41.5%) or senior physicians (*n* = 121; 37.5%), followed by consultants (*n* = 49; 15.2%), and head physicians (*n* = 19; 5.9%). As expected, professional surgical experience differed significantly across these hierarchical groups: the majority of resident physicians (*n* = 102; 76.1%) had less than five years of experience, while all head physicians reported more than 15 years (*p* < 0.001). Previous use of AI was prevalent: 58.5% (*n* = 189) of respondents reported occasional use, while 28.2% (*n* = 91) reported regular use (Fig. 2A). The most common areas of AI application were speech recognition (*n* = 211; 65.3%) and chatbots/large language models (*n* = 203; 62.8%), followed by literature analysis/evidence mining (*n* = 129; 39.9%), and image-based diagnostics (*n* = 128; 39.6%). Only 6.5% of respondents (*n* = 21) reported no prior use of AI (Fig. 2B). The application patterns varied depending on the level of surgical training for selected domains: surgical planning and image-guided surgery were significantly more frequent among head physicians (*p* < 0.001 and *p* = 0.009, respectively). The respondents’ self-rated AI knowledge was predominantly classified as “basic” (*n* = 134; 41.5%) and “average” (*n* = 129; 39.9%), while 14.2% rated themselves as “above average” (*n* = 46) and 1.2% as “experts” (*n* = 4), as shown in Fig. 3. Data is also shown in Supplementary Table 2.

### Surgeons’ anticipated benefits of AI in patient care and beyond

**Fig. 4:**
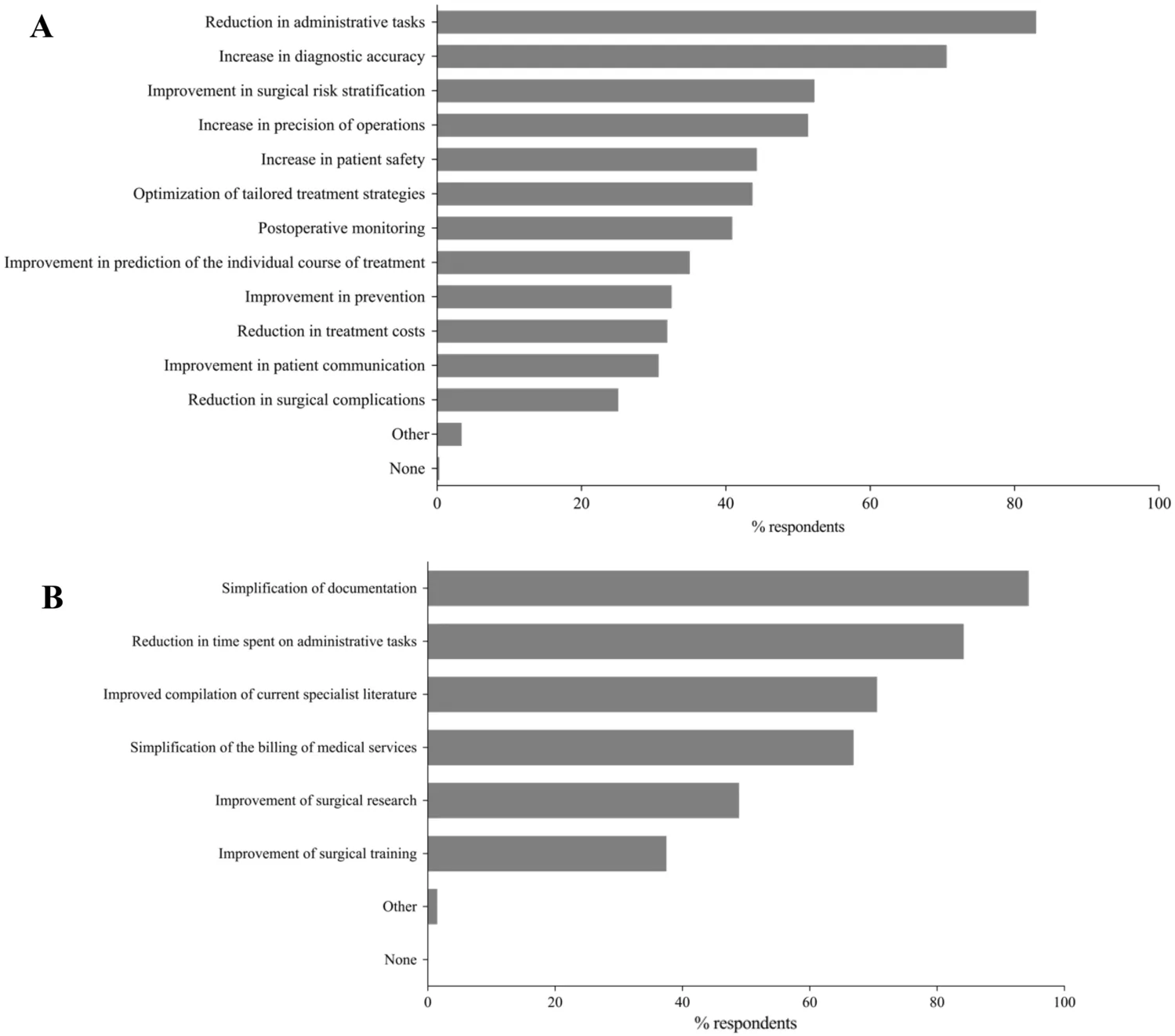
**A** Benefits of AI expected by respondents within, and **B** outside of patient care; multiple selections possible

In terms of patient care, the most frequently anticipated benefits of AI were a reduction in administrative tasks (*n* = 268; 83.0%) and an increase in diagnostic accuracy (*n* = 228; 70.6%), as shown in Fig. 4A. Slightly more than half of the respondents anticipated improvements in surgical risk stratification (*n* = 169; 52.3%) and an increase in precision of operations (*n* = 166; 51.4%), followed by an increase in patient safety (*n* = 143; 44.3%), optimization of tailored treatment strategies (*n* = 141; 43.7%), and postoperative monitoring (*n* = 132; 40.9%). The selection of optimization of tailored treatment strategies increased significantly with increasing professional experience, with the highest value being recorded among head physicians (*n* = 14; 73.7%; *p* = 0.003). With regard to the potential of AI outside the domain of patient care, the simplification of documentation was mentioned most frequently (*n* = 305; 94.4%), as shown in Fig. 4B. Head physicians showed significantly lower approval ratings for simplifying documentation (*n* = 15; 78.9%; *p* = 0.007). Moreover, the majority of respondents reported a reduction in time spent on administrative tasks (*n* = 272; 84.2%), while an improved compilation of current specialist literature (*n* = 228; 70.6%) and a simplified billing process for medical services (*n* = 216; 66.9%) were also commonly cited benefits. Almost half of the respondents anticipated benefits for surgical research (*n* = 158; 48.9%), while over one-third anticipated benefits for surgical training (*n* = 121; 37.5%). Data is also shown in Supplementary Table 3.

### AI-based chatbots in surgical practice

**Fig. 5:**
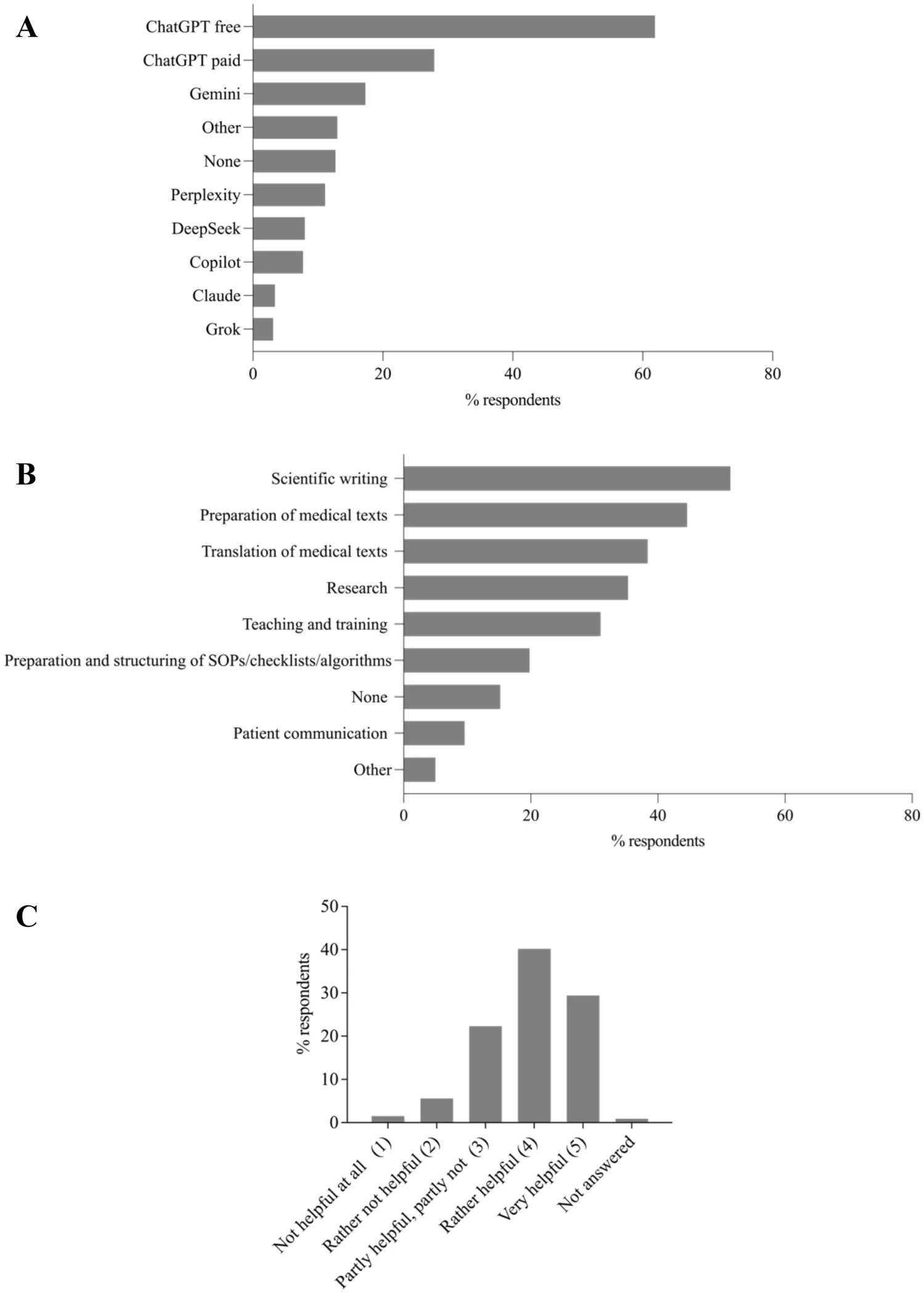
**A** Use of AI-based chatbots among respondents, **B** their applications, and **C** assessment of the usefulness of chatbots in daily surgical practice; multiple selections were possible for **A** and **B**

Among the respondents, the free version of ChatGPT (*n* = 200; 61.9%) from OpenAI (USA) dominated chatbot usage, followed by its paid version (*n* = 90; 27.9%), as shown in Fig. 5A. Other chatbots were used less frequently: Gemini (*n* = 56; 17.3%) from Google (USA), Perplexity (*n* = 36; 11.1%) from Perplexity AI (USA), DeepSeek (*n* = 26; 8.0%) from Hangzhou DeepSeek Artificial Intelligence (China), and Copilot (*n* = 25; 7.7%) from Microsoft (USA). The use of other chatbot platforms not mentioned in the present survey was reported by 13% of respondents (*n* = 42), while 12.7% reported not using any (*n* = 41). The use of Microsoft Copilot and Google Gemini was reported significantly more frequently by head physicians (*p* = 0.006 and *p* = 0.044, respectively), while alternative chatbot platforms not referenced in this survey were reported at a significantly elevated frequency by resident physicians (*p* = 0.020). With regard to the applications of AI-based chatbots, the most common use case was scientific writing (*n* = 166; 51.4%) with slightly more than half of respondents, followed by the preparation of medical texts (*n* = 144; 44.6%), translation (*n* = 124; 38.4%), research (*n* = 114; 35.3%), and teaching/training (*n* = 100; 31.0%), as shown in Fig. 5B. Across increasing surgical training levels, a significant downward trend was observed in the preparation of medical texts (resident physicians 50.7% vs. head physicians 31.6%; *p* = 0.019) and in scientific writing (55.2 vs. 31.6%; *p* = 0.038). The majority of respondents (*n* = 225; 69.6%) rated the use of AI chatbots in daily surgical practice positively (Fig. 5C). Of these, 40.2% considered them “rather helpful” (*n* = 130) and 29.4% “very helpful” (*n* = 95). Data is also shown in Supplementary Table 4.

### Barriers and requirements for routine AI adoption

**Fig. 6:**
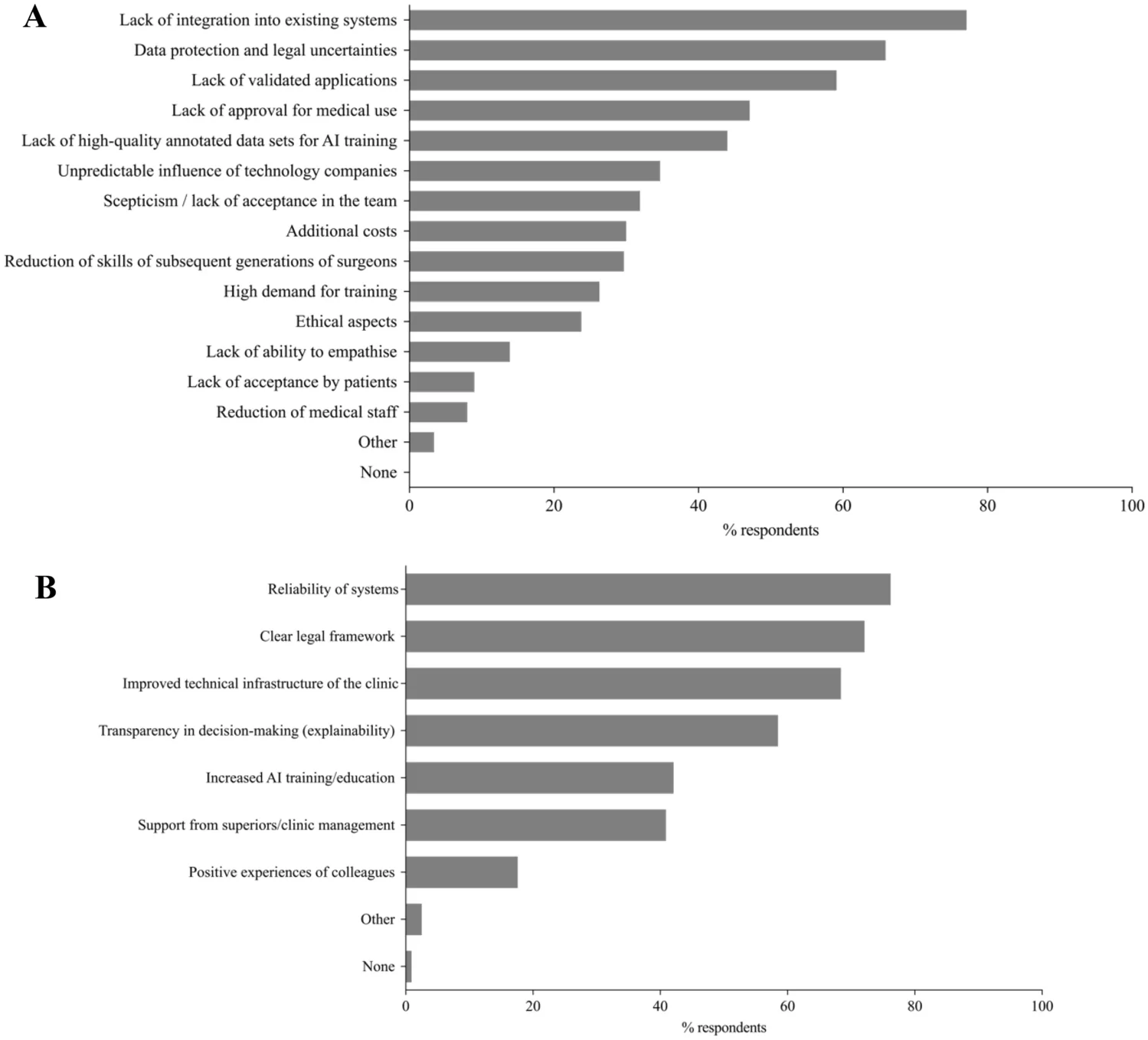
**A** Perceived barriers to the routine use of AI in surgery, and **B** requirements for its adoption; multiple selections possible

The most frequently reported barriers to routine AI use in surgery were lack of integration into existing systems (*n* = 249; 77.1%), followed by data-protection and legal uncertainties (*n* = 213; 65.9%), and lack of validated applications (*n* = 191; 59.1%), as shown in Fig. 6A. Almost half of the respondents anticipated additional barriers in lack of approval for medical use (*n* = 152; 47.1%), and lack of high-quality annotated data sets for AI training (*n* = 142; 44.0%). The concern that AI could impair the skills of subsequent generations of surgeons was reported by 29.7% (*n* = 96) of respondents and increased significantly with advancing levels of surgical training (*p* = 0.034). Additional costs due to the use of AI were reported by 30% (*n* = 97) of respondents, while head physicians cited this as a barrier significantly more often than resident physicians (*χ*^2^ *p* = 0.010; trend *p* = 0.026). A limited ability to empathize (*n* = 45; 13.9%), a lack of acceptance by patients (*n* = 29; 9.0%), and the possibility for medical staff reductions (*n* = 26; 8.0%) were mentioned less frequently. Notably, no respondent selected “none”. The requirements for the routine adoption of AI focused on the reliability of systems (*n* = 246; 76.2%), a clear legal framework (*n* = 233; 72.1%), improved technical infrastructure in clinics (*n* = 221; 68.4%), and transparency in decision-making (*n* = 189; 58.5%), as shown in Fig. 6B. In addition, increased AI training/education (*n* = 136; 42.1%) and support from superiors/clinic management (*n* = 132; 40.9%) were also frequently requested. Compared to head physicians, resident physicians significantly more frequently requested improved technical infrastructure (77.6 vs. 57.9%; *χ*^2^ *p* = 0.019; trend *p* = 0.002) and support from superiors/clinic management (50.7 vs. 21.1%; *χ*^2^ *p* = 0.012; trend *p* = 0.001). Data is also shown in Supplementary Table 5.

### Potential and future prospects for the use of AI

**Fig. 7:**
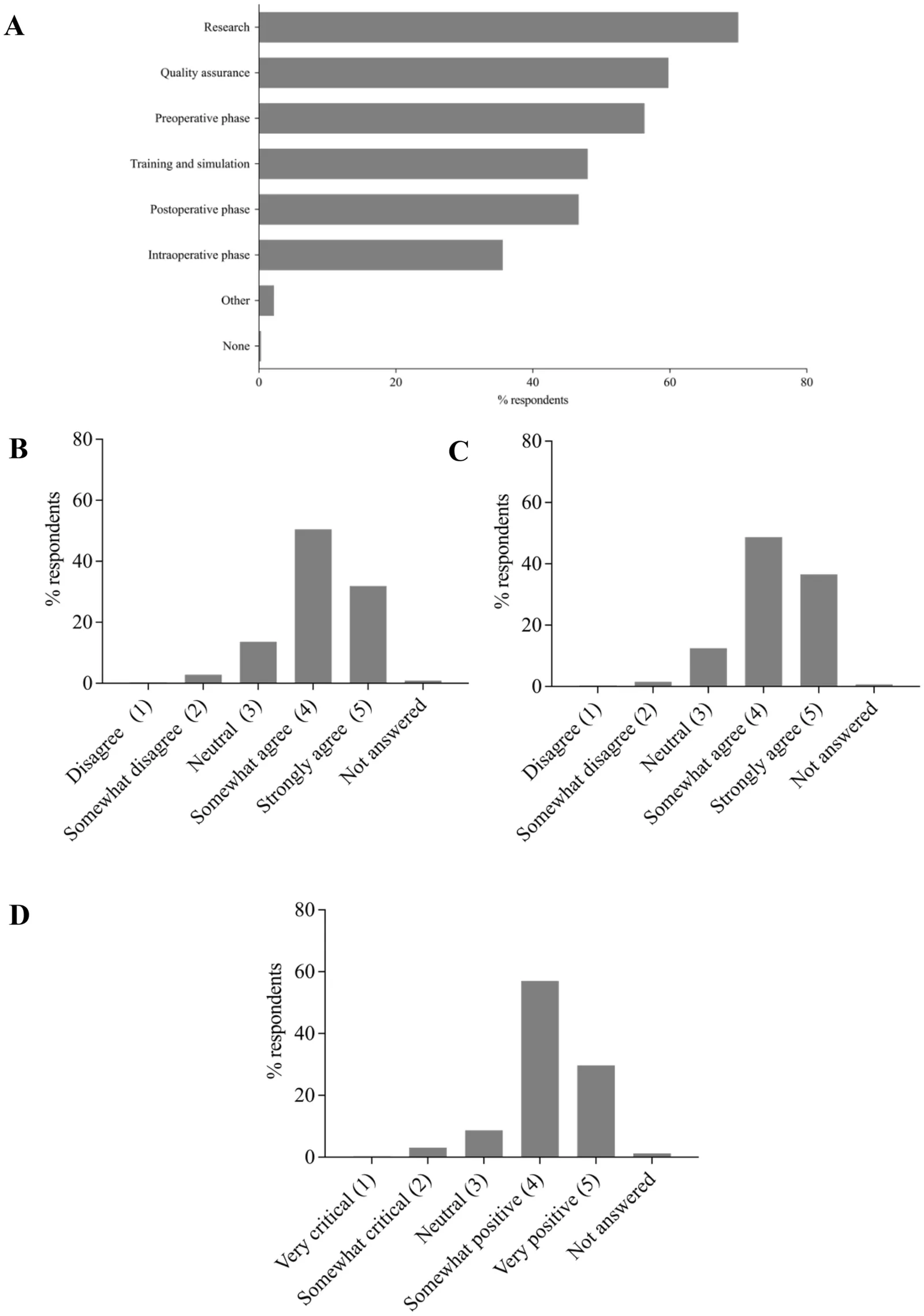
**A** Potential for practical application of AI in surgery in the near future, **B** assessment of improvement in surgical care quality through AI, **C** need for structured AI training, and **D** 5-year outlook regarding the use of AI in surgery; multiple selections were possible for **A**

With regard to the present most promising potential for the practical application of AI in surgery in the near future, the majority of respondents reported research (*n* = 226; 70.0%), as shown in Fig. 7A. In addition, more than half of the respondents endorsed quality assurance (*n* = 193; 59.8%) and the preoperative phase (*n* = 182; 56.3%). Training and simulation (*n* = 155; 48.0%) and the postoperative phase (*n* = 151; 46.7%) were selected by almost half of the respondents, while intraoperative phase was selected less frequently (*n* = 115; 35.6%). For the intraoperative phase, there was a significant higher endorsement with increasing surgical training level (*p* = 0.015). A contrary trend was observed for the “other” category, which was significantly most frequently cited by resident physician (*p* = 0.036). The majority of respondents (*n* = 266; 82.4%) anticipated that AI will improve the quality of surgical care, with 31.9% of these “strongly agree” with this statement (*n* = 103), as shown in Fig. 7B. In addition, a clear majority (*n* = 275; 85.1%) endorsed the need for structured AI training (Fig. 7C). The 5-year outlook regarding the utilization of AI in surgery was predominantly positive (*n* = 280; 86.7%), as shown in Fig. 7D. Data is also shown in the Supplemental Material Table 6.

## Discussion

In our study cohort of 323 surgeons, predominantly resident and senior physicians, self-reported AI use was frequent, indicating that respondents from both newer and more experienced surgical generations are engaging with AI. However, usage was mainly concentrated on low-threshold tools such as speech recognition and chatbots, which suggests an early stage of AI adoption rather than deep workflow integration. The hierarchical signal in our data, with head physicians reporting significantly greater engagement in more advanced AI domains (e.g. surgical planning and image-guided surgery), indicates that implementation may be driven “top-down” where institutional resources and leadership are required. Although AI exposure was high in our cohort, AI literacy remained modest: 41.5% self-rated their knowledge as “basic” and 39.9% as “average”, while only 14.2% reported “above average” and 1.2% “expert” knowledge. This “adoption-before-literacy” is consistent with previous German and international surveys, suggesting that many surgeons interact with AI without feeling competent enough to evaluate its performance, biases, or failure modes [14–18].

Surgeons in our study cohort primarily anticipated near-term, workflow-oriented gains, with the leading expected benefits in patient care being a reduction in administrative tasks and improved diagnostic accuracy. Interest in more “high-stakes” clinical support was present but less dominant, as only about half expected improvements in surgical risk stratification and operative precision. Outside of patient care, expectations were even more pronounced for documentation simplification and reduced administrative time, followed by literature compilation and billing. These findings reflect perceptions rather than evidence of improved clinical performance, as objective outcomes, workflow efficiency metrics, patient outcomes, and safety endpoints were not assessed. Overall, surgeons appear to view AI primarily as an efficiency-enhancing tool rather than as a substitute for clinical decision-making. This was confirmed by prior surveys: Kinross et al. reported that British colorectal surgeons ranked documentation and imaging as key domains of AI [17], while Pecqueux et al. demonstrated that German surgeons expressed greater confidence in AI for diagnostic purposes than for therapeutic decision-making [14]. Furthermore, a large international survey by Arboit et al. among 671 respondents from 98 countries revealed that surgeons valued AI for administrative and management functions [18]. Interestingly, our study confirmed a seniority effect: head physicians significantly prioritized AI for tailored treatment strategies and showed lower approval for documentation simplification. This phenomenon may be indicative of divergent task distributions, suggesting that senior roles prioritize AI-supported strategic decision-making.

In our study cohort, AI-based chatbots were frequently used by respondents. ChatGPT from OpenAI (USA) emerged as the predominant platform of choice, and approximately 70% of participating surgeons regarded chatbots as beneficial. This indicates that chatbots have become a commonly used tool among respondents, primarily for text-based productivity tasks. In particular, the reported use was focused on text-centric tasks such as scientific writing and preparing medical texts, suggesting that chatbots are currently being adopted primarily as productivity and communication tools rather than as “clinical decision engines”. This “productivity-first” approach is consistent with other surveys, in which clinicians prioritize documentation and information-handling tasks [8, 17–19]. Moreover, the potential for chatbots to provide immediate benefits in the domains of patient-facing documentation and education is already evident [6, 20, 21]. However, current evidence also underscores significant limitations. Accuracy and reliability are major concerns: AI-based chatbots may generate incorrect information (“hallucinations”) and, depending on the prompt, model, and version used, may provide references that are incomplete, difficult to verify, or fabricated; therefore, their outputs require careful expert review [22, 23]. The efficacy of structured prompting strategies, such as explicitly requesting sources, uncertainty statements, or a stated level of evidence, in reducing unsupported outputs remains to be evaluated. Future studies should focus on this aspect, as it was not included in the present survey. Readability and personalization are often suboptimal, and users have noted that many chatbot responses lack a clear evidence base [24]. In addition, ethical and governance issues arise, and clear guidelines are needed to define accountability and patient‐privacy safeguards when using AI in patient care [16].

The present survey identified insufficient system integration, legal and data-protection uncertainty, and limited availability of validated applications as key barriers to the routine adoption of AI into surgical workflows. Concerns about skill degradation in future generations of surgeons and additional costs were reported significantly more often by head physicians and highlight that cultural and economic factors remain relevant alongside technical barriers. As our questionnaire did not ask which specific skills respondents feared might deteriorate, this finding should be interpreted as a general concern rather than being assigned to particular technical or cognitive domains. Moreover, the reasons for hierarchical group-specific differences in AI use, including lower uptake in some consultant-level analyses, were not assessed by our questionnaire and therefore remain uncertain. Although lack of integration was the most frequently selected barrier, our questionnaire did not assess where current AI tools are situated within the surgical workflow or how integration problems manifest in day-to-day practice. The present findings are consistent with the results of previous surveys, which identified limited AI literacy, unresolved liability, and trust gaps as significant barriers to implementation [14, 16–19, 25]. At the same time, hospitals should establish formal AI governance structures rather than ad-hoc tool deployment, such as clinical ownership, data stewardship, and incident reporting, to ensure safe organizational implementation [26–28]. Surgeons participating in the present survey prioritized reliability, a clear legal framework, infrastructure, and transparency as the most important requirements for AI adoption. Consequently, the procurement of AI systems should necessitate external validation, calibration reports, audit trails and explainability appropriate to the surgical task in order to avoid general “black box” assurances. On the regulatory side, the EU AI Act (in force since August 2024) provides for a risk-based compliance pathway and emphasizes governance—elements that directly correspond to the medico-legal uncertainty reported by respondents [29].

In our study cohort, surgeons perceived the most immediate AI potential in research, quality assurance, and the preoperative phase, whereas intraoperative applications were less frequently prioritized. This pattern suggests that surgeons anticipate more rapid returns from AI that scales across many patients than from technically complex “operation room (OR)-assistance,” such as information synthesis, risk prediction, and quality metrics. The increasing acceptance of intraoperative AI with rising surgical training levels indicates that OR-facing systems may be driven by senior surgeons, who also influence governance and investment decisions. The majority of respondents expected AI to improve the quality of surgical care (82.4%), and gave a positive 5-year outlook (86.7%). This optimism aligns with international trends: a recent global survey found that nearly 80% of surgeons expect AI to improve surgical practice, despite ongoing knowledge gaps and infrastructural barriers to implementation [18]. However, these positive AI expectations are likely to remain dependent on validation and suitability for surgical workflow, and do not imply readiness for autonomous systems. Future work should therefore focus less on isolated task automation and more on embedding AI into validated, closed-loop clinical workflows. The strong demand for structured AI training in our survey (85.1%) indicates that surgeons recognize a competency gap that could otherwise widen as tools diffuse faster than formal education.

### Limitations

The present study has several limitations. First, the response rate was low (8.7%), which raises substantial concern for non-response bias. Because recruitment focused on university hospitals, selection bias is likely and generalizability to non-academic settings is limited. Research-oriented academic surgeons already interested in AI may have been more likely to participate, potentially overestimating AI use, optimism, and chatbot adoption. The study design does not allow for any conclusions regarding the comparative use of advanced AI tools in academic and non-academic settings. Second, the sample size (*n* = 323) is not representative of all regions in Germany, Austria, and Switzerland. Third, the questionnaire was an exploratory 16-item instrument developed for this study and was not formally pilot-tested or psychometrically validated; therefore, reliability and construct validity cannot be assumed. Fourth, voluntary participation may have introduced response bias, with surgeons interested in AI more likely to respond, potentially overestimating AI use and optimism. Fifth, all outcomes were self-reported and therefore prone to subjective interpretation and recall bias. Moreover, the study did not measure objective workflow effects, patient outcomes, or safety endpoints and thus cannot determine whether perceived benefits translate into real-world clinical benefit. Sixth, the questionnaire did not capture the hardware or institutional context of AI use, such as whether access occurred via smartphone or hospital computer, or whether respondents’ institutions provided formal AI education, local tools, or implementation support. Finally, common-method bias cannot be excluded, as participants may have inferred the survey’s intent and adapted their responses accordingly [30].

## Conclusion

The use of artificial intelligence, particularly speech recognition and LLM-based chatbots, was commonly reported by general surgeons in German-speaking countries, primarily for documentation, scientific writing, and other text-based productivity tasks rather than validated clinical decision support or intraoperative use. To enable broader AI adoption, surgeons highlighted the need for interoperable infrastructure, externally validated clinical-grade applications, legal and data-protection clarity, and structured AI education. Future work should focus on embedding AI within validated, closed-loop clinical workflows rather than on isolated task automation.

## Supplementary Information

Below is the link to the electronic supplementary material.

Supplementary material 1 (DOCX 24 kb)

Supplementary material 2 (DOCX 22 kb)

Supplementary material 3 (DOCX 24 kb)

Supplementary material 4 (DOCX 23 kb)

Supplementary material 5 (DOCX 27 kb)

Supplementary material 6 (DOCX 23 kb)
