# Supplementary material for: Artificial intelligence and chatbots in general surgery: a survey among surgeons in Germany, Austria and Switzerland
Source: Surg Endosc. 2026 Jul 1;40(9):7473–83. doi: 10.1007/s00464-026-13044-5 (PMC13582102; doi:10.1007/s00464-026-13044-5)
Supplement: Supplementary file 1 [file 464_2026_13044_MOESM1_ESM.docx]

**Supplementary Table 1** Questionnaire details. English translation of the questionnaire used in the survey. The survey was originally designed and distributed in German. AI, artificial intelligence; SOP, standard operating procedure

***Survey Questionnaire***

**1. Which position applies best to you?**

- Resident
- Consultant
- Senior physician
- Head physician

**2. How many years of professional experience in surgery do you have?**

- < 5 years
- 5 - 10 years
- 11 - 15 years
- > 15 years

**3. Have you previously encountered artificial intelligence (AI) in your professional life?**

- Yes, regularly
- Yes, occasionally
- No, not yet

**4. In which area(s) have you previously employed or encountered AI? (Multiple choice)**

- Image diagnostics (e.g. CT/MRI interpretation, endoscopy)
- Surgical planning (e.g. 3D models)
- Image-guided surgery (augmented reality)
- Assistance systems for minimally invasive/robotic surgery
- Chatbots/Large Language Models (e.g. ChatGPT)
- Automated documentation
- Speech recognition (e.g. surgical reports)
- Decision support systems (e.g. prediction models for complications, therapy response, outcome)
- Postoperative monitoring
- Training (e.g. virtual surgery simulation)
- Literature analysis/evidence mining
- Other
- None

**5. How would you rate your own knowledge of AI in a medical context?**

- No knowledge
- Rudimentary knowledge
- Average knowledge
- Above average knowledge
- Expert knowledge

**6. In which area(s) of patient care do you perceive the greatest potential for AI? (Multiple choice)**

- Increase in diagnostic accuracy
- Improvement in prevention
- Improvement in surgical risk stratification
- Improvement in prediction of the individual course of treatment
- Improvement in patient communication
- Optimization of tailored treatment strategies
- Increase in patient safety
- Postoperative monitoring (e.g. vital signs)
- Reduction in surgical complications
- Reduction in administrative tasks
- Reduction in treatment costs
- Increase in precision of operations (e.g. augmented reality, navigation)
- Other
- None

**7. In which area(s) outside patient care do you perceive the greatest potential for AI? (Multiple choice)**

- Simplification of documentation (e.g. doctor's letters, surgical reports)
- Simplification of the billing of medical services (e.g. German DRG system)
- Reduction in time spent on administrative tasks
- Improved compilation of current specialist literature (e.g. research via chatbot)
- Improvement of surgical training
- Improvement of surgical research
- Other
- None

**8. AI can improve the quality of surgical care.**

- Disagree
- Rather disagree
- Neutral
- Rather agree
- Totally agree

**9. What barrier(s) exist that currently hinder the broader utilisation of AI in surgical procedures? (Multiple choice)**

- Lack of validated applications
- Lack of high-quality annotated data sets for AI training
- Lack of integration into existing systems
- Data protection and legal uncertainties
- Ethical aspects
- Scepticism / lack of acceptance in the team
- High demand for training
- Lack of acceptance by patients
- Lack of ability to empathise
- Reduction of medical staff
- Additional costs
- Lack of approval for medical field
- Unpredictable influence of technology companies
- Reduction of skills of subsequent generations of surgeons
- Other
- None

**10. I would like to learn more about AI in training courses.**

- Disagree
- Rather disagree
- Neutral
- Rather agree
- Totally agree

**11. Which chatbot/large language model (LLM) do you use professionally? (Multiple choice)**

- ChatGPT free (OpenAI)
- ChatGPT chargeable (OpenAI)
- Claude (Anthropic)
- Copilot (Microsoft)
- DeepSeek (High-Flyer)
- Gemini (Google)
- Grok (xAI)
- Perplexity
- Other
- None

**12. In which area(s) do you use AI chatbots in your surgical working environment? (Multiple choice)**

- Preparation of medical texts (e.g. doctor's letters, surgical reports)
- Scientific writing (e.g. publications, study protocols, ethics applications)
- Research (e.g. data analysis)
- Teaching and training (e.g. case studies, presentations, examination questions)
- Patient communication (e.g. texts written in layman's terms, surgical information)
- Preparation and structuring of SOPs, checklists or treatment algorithms
- Translation of medical texts (e.g. for international patients)
- Other
- None

**13. In your opinion, how helpful is the use of AI chatbots in everyday surgery?**

- Not helpful at all
- Rather not helpful
- Partly helpful, partly not
- Rather helpful
- Very helpful

**14. Where do you currently see the greatest potential for the practical use of AI in surgery in the near future? (Multiple choice)**

- Preoperative phase (e.g. diagnostics, therapy decision, surgical planning, prognosis)
- Intraoperative phase (e.g. robotics, navigation, image guidance, real-time analysis, perfusion status)
- Postoperative phase (e.g. complication detection, progression prognoses, automated documentation)
- Training and simulation
- Research (e.g. literature analysis, evidence mining, data analysis)
- Quality assurance (e.g. big data analyses)
- Other
- None

**15. What requirement(s) would have to be met for you to use AI in everyday surgery? (Multiple choice)**

- Reliability of systems
- Transparency of decisions (explainability)
- Increased AI training/education
- Clear legal framework
- Positive experiences of colleagues
- Improved technical infrastructure of the clinic
- Support from superiors/clinic management
- Other
- None

**16. What is your personal view on the use of AI in surgery in the next 5 years?**

- Very critical
- Rather critical
- Neutral
- Rather positive
- Very positive
