# Supplementary material for: Artificial intelligence and chatbots in general surgery: a survey among surgeons in Germany, Austria and Switzerland
Source: Surg Endosc. 2026 Jul 1;40(9):7473–83. doi: 10.1007/s00464-026-13044-5 (PMC13582102; doi:10.1007/s00464-026-13044-5)
Supplement: Supplementary file 2 [file 464_2026_13044_MOESM2_ESM.docx]

**Supplementary Table 2** Demographic data and respondents' previous experiences with AI

| **Characteristic** | **Overall**  N = 323^1^ | **Resident physician** n=134 (41.5%)^1^ | **Consultant** n=49 (15.2%)^1^ | **Senior physician** n=121 (37.5%)^1^ | **Head physician** n=19 (5.9%)^1^ | **p-value**^2^ |
| --- | --- | --- | --- | --- | --- | --- |
| **Professional experience (years)** |  |  |  |  |  | **<0.001** |
| < 5 | 102 (31.6%) | 102 (76.1%) | 0 (0.0%) | 0 (0.0%) | 0 (0.0%) |  |
| 5 - 10 | 74 (22.9%) | 30 (22.4%) | 31 (63.3%) | 13 (10.7%) | 0 (0.0%) |  |
| 11 - 15 | 61 (18.9%) | 2 (1.5%) | 12 (24.5%) | 47 (38.8%) | 0 (0.0%) |  |
| > 15 | 86 (26.6%) | 0 (0.0%) | 6 (12.2%) | 61 (50.4%) | 19 (100.0%) |  |
| **Experiences with AI** |  |  |  |  |  | 0.659 |
| No, not yet | 43 (13.3%) | 20 (14.9%) | 8 (16.3%) | 14 (11.6%) | 1 (5.3%) |  |
| Yes, occasionally | 189 (58.5%) | 75 (56.0%) | 28 (57.1%) | 76 (62.8%) | 10 (52.6%) |  |
| Yes, regularly | 91 (28.2%) | 39 (29.1%) | 13 (26.5%) | 31 (25.6%) | 8 (42.1%) |  |
| **Previous use of AI** |  |  |  |  |  |  |
| Image diagnostics (e.g. CT/MRI interpretation, endoscopy) | 128 (39.6%) | 52 (38.8%) | 12 (24.5%) | 54 (44.6%) | 10 (52.6%) | 0.062 |
| Surgical planning (e.g. 3D models) | 57 (17.6%) | 11 (8.2%) | 8 (16.3%) | 32 (26.4%) | 6 (31.6%) | **<0.001** |
| Image-guided surgery (e.g. augmented reality) | 40 (12.4%) | 15 (11.2%) | 4 (8.2%) | 14 (11.6%) | 7 (36.8%) | **0.009** |
| Assistance systems for minimally invasive/robotic surgery | 51 (15.8%) | 21 (15.7%) | 5 (10.2%) | 18 (14.9%) | 7 (36.8%) | 0.056 |
| Chatbots/Large Language Models (e.g. ChatGPT) | 203 (62.8%) | 91 (67.9%) | 30 (61.2%) | 71 (58.7%) | 11 (57.9%) | 0.453 |
| Automated documentation | 51 (15.8%) | 23 (17.2%) | 5 (10.2%) | 18 (14.9%) | 5 (26.3%) | 0.392 |
| Speech recognition (e.g. surgical reports) | 211 (65.3%) | 83 (61.9%) | 30 (61.2%) | 85 (70.2%) | 13 (68.4%) | 0.491 |
| Decision support systems (e.g. prediction models for complications, therapy response, outcome) | 34 (10.5%) | 12 (9.0%) | 5 (10.2%) | 14 (11.6%) | 3 (15.8%) | 0.788 |
| Postoperative monitoring | 7 (2.2%) | 2 (1.5%) | 1 (2.0%) | 2 (1.7%) | 2 (10.5%) | 0.082 |
| Training (e.g. virtual surgery simulation) | 65 (20.1%) | 29 (21.6%) | 5 (10.2%) | 25 (20.7%) | 6 (31.6%) | 0.190 |
| Literature analysis/evidence mining | 129 (39.9%) | 56 (41.8%) | 22 (44.9%) | 44 (36.4%) | 7 (36.8%) | 0.702 |
| Other | 25 (7.7%) | 11 (8.2%) | 6 (12.2%) | 8 (6.6%) | 0 (0.0%) | 0.356 |
| None | 21 (6.5%) | 9 (6.7%) | 6 (12.2%) | 5 (4.1%) | 1 (5.3%) | 0.280 |
| **Own knowledge of AI** |  |  |  |  |  | 0.121 |
| No knowledge (1) | 6 (1.9%) | 2 (1.5%) | 1 (2.0%) | 3 (2.5%) | 0 (0.0%) |  |
| Basic knowledge (2) | 134 (41.5%) | 59 (44.0%) | 24 (49.0%) | 50 (41.3%) | 1 (5.3%) |  |
| Average knowledge (3) | 129 (39.9%) | 51 (38.1%) | 17 (34.7%) | 51 (42.1%) | 10 (52.6%) |  |
| Above average knowledge (4) | 46 (14.2%) | 18 (13.4%) | 6 (12.2%) | 15 (12.4%) | 7 (36.8%) |  |
| Expert knowledge (5) | 4 (1.2%) | 3 (2.2%) | 0 (0.0%) | 1 (0.8%) | 0 (0.0%) |  |
| [NA] Not answered | 4 (1.2%) | 1 (0.7%) | 1 (2.0%) | 1 (0.8%) | 1 (5.3%) |  |
| ^1^n (%) | | | | | | |
| ^2^Pearson's Chi-squared test | | | | | | |
