# Supplementary material for: Artificial intelligence and chatbots in general surgery: a survey among surgeons in Germany, Austria and Switzerland
Source: Surg Endosc. 2026 Jul 1;40(9):7473–83. doi: 10.1007/s00464-026-13044-5 (PMC13582102; doi:10.1007/s00464-026-13044-5)
Supplement: Supplementary file 3 [file 464_2026_13044_MOESM3_ESM.docx]

**Supplementary Table 3** Potential use of AI within and outside patient care

| **Characteristic** | | **Overall**  N = 323^1^ | **Resident physician** n=134 (41.5%)^1^ | **Consultant** n=49 (15.2%)^1^ | **Senior physician** n=121 (37.5%)^1^ | **Head physician** n=19 (5.9%)^1^ | **p-value** |
| --- | --- | --- | --- | --- | --- | --- | --- |
| **Within patient care** | | | | | | | |
|  | Increase in diagnostic accuracy | 228 (70.6%) | 93 (69.4%) | 37 (75.5%) | 83 (68.6%) | 15 (78.9%) | 0.675 |
|  | Improvement in prevention | 105 (32.5%) | 50 (37.3%) | 11 (22.4%) | 39 (32.2%) | 5 (26.3%) | 0.261 |
|  | Improvement in surgical risk stratification | 169 (52.3%) | 65 (48.5%) | 25 (51.0%) | 66 (54.5%) | 13 (68.4%) | 0.387 |
|  | Improvement in prediction of the individual course of treatment | 113 (35.0%) | 41 (30.6%) | 14 (28.6%) | 48 (39.7%) | 10 (52.6%) | 0.122 |
|  | Improvement in patient communication | 99 (30.7%) | 35 (26.1%) | 19 (38.8%) | 38 (31.4%) | 7 (36.8%) | 0.363 |
|  | Optimization of tailored treatment strategies | 141 (43.7%) | 47 (35.1%) | 19 (38.8%) | 61 (50.4%) | 14 (73.7%) | **0.003** |
|  | Increase in patient safety | 143 (44.3%) | 57 (42.5%) | 17 (34.7%) | 57 (47.1%) | 12 (63.2%) | 0.163 |
|  | Postoperative monitoring (e.g. vital signs) | 132 (40.9%) | 56 (41.8%) | 17 (34.7%) | 50 (41.3%) | 9 (47.4%) | 0.762 |
|  | Reduction in surgical complications | 81 (25.1%) | 38 (28.4%) | 9 (18.4%) | 26 (21.5%) | 8 (42.1%) | 0.127 |
|  | Reduction in administrative tasks | 268 (83.0%) | 112 (83.6%) | 41 (83.7%) | 100 (82.6%) | 15 (78.9%) | 0.964 |
|  | Reduction in treatment costs | 103 (31.9%) | 46 (34.3%) | 13 (26.5%) | 36 (29.8%) | 8 (42.1%) | 0.535 |
|  | Increase in precision of operations (e.g. augmented reality, navigation) | 166 (51.4%) | 60 (44.8%) | 24 (49.0%) | 69 (57.0%) | 13 (68.4%) | 0.102 |
|  | Other | 11 (3.4%) | 4 (3.0%) | 2 (4.1%) | 4 (3.3%) | 1 (5.3%) | 0.952 |
|  | None | 1 (0.3%) | 0 (0.0%) | 0 (0.0%) | 1 (0.8%) | 0 (0.0%) | 0.643 |
| **Outside patient care** | | | | | | | |
|  | Simplification of documentation (e.g. doctor's letters, surgical reports) | 305 (94.4%) | 128 (95.5%) | 49 (100.0%) | 113 (93.4%) | 15 (78.9%) | **0.007** |
|  | Simplification of the billing of medical services (e.g. German DRG system) | 216 (66.9%) | 95 (70.9%) | 29 (59.2%) | 78 (64.5%) | 14 (73.7%) | 0.391 |
|  | Reduction in time spent on administrative tasks | 272 (84.2%) | 115 (85.8%) | 39 (79.6%) | 103 (85.1%) | 15 (78.9%) | 0.678 |
|  | Improved compilation of current specialist literature (e.g. research via chatbot) | 228 (70.6%) | 97 (72.4%) | 36 (73.5%) | 80 (66.1%) | 15 (78.9%) | 0.530 |
|  | Improvement of surgical training | 121 (37.5%) | 48 (35.8%) | 18 (36.7%) | 46 (38.0%) | 9 (47.4%) | 0.807 |
|  | Improvement of surgical research | 158 (48.9%) | 69 (51.5%) | 20 (40.8%) | 58 (47.9%) | 11 (57.9%) | 0.512 |
|  | Other | 5 (1.5%) | 2 (1.5%) | 1 (2.0%) | 1 (0.8%) | 1 (5.3%) | 0.529 |
|  | None | 0 (0.0%) | 0 (0.0%) | 0 (0.0%) | 0 (0.0%) | 0 (0.0%) |  |

^1^n (%)
