# Supplementary material for: Artificial intelligence and chatbots in general surgery: a survey among surgeons in Germany, Austria and Switzerland
Source: Surg Endosc. 2026 Jul 1;40(9):7473–83. doi: 10.1007/s00464-026-13044-5 (PMC13582102; doi:10.1007/s00464-026-13044-5)
Supplement: Supplementary file 4 [file 464_2026_13044_MOESM4_ESM.docx]

**Supplementary Table 4** Use of AI-based chatbot versions, their application areas in surgery and assessment of the usefulness; SOP, standard operating procedure

| **Characteristic** | **Overall**  N = 323^1^ | **Resident physician** n=134 (41.5%)^1^ | **Consultant** n=49 (15.2%)^1^ | **Senior physician** n=121 (37.5%)^1^ | **Head physician** n=19 (5.9%)^1^ | **p-value**^2^ | **p-value**^3^ |
| --- | --- | --- | --- | --- | --- | --- | --- |
| **Assessment of chatbot usefulness** |  |  |  |  |  | 0.114 | **0.017** |
| Not helpful at all | 5 (1.5%) | 1 (0.7%) | 1 (2.0%) | 3 (2.5%) | 0 (0.0%) |  |  |
| Rather not helpful | 18 (5.6%) | 6 (4.5%) | 1 (2.0%) | 9 (7.4%) | 2 (10.5%) |  |  |
| Partly helpful, partly not | 72 (22.3%) | 26 (19.4%) | 16 (32.7%) | 30 (24.8%) | 0 (0.0%) |  |  |
| Rather helpful | 130 (40.2%) | 50 (37.3%) | 17 (34.7%) | 52 (43.0%) | 11 (57.9%) |  |  |
| Very helpful | 95 (29.4%) | 50 (37.3%) | 14 (28.6%) | 25 (20.7%) | 6 (31.6%) |  |  |
| [NA] Not answered | 3 (0.9%) | 1 (0.7%) | 0 (0.0%) | 2 (1.7%) | 0 (0.0%) |  |  |
| **Chatbot version** |  |  |  |  |  |  |  |
| ChatGPT free version (OpenAI) | 200 (61.9%) | 89 (66.4%) | 29 (59.2%) | 70 (57.9%) | 12 (63.2%) | 0.538 | 0.234 |
| ChatGPT paid version (OpenAI) | 90 (27.9%) | 37 (27.6%) | 13 (26.5%) | 36 (29.8%) | 4 (21.1%) | 0.873 | 0.997 |
| Claude (Anthropic) | 11 (3.4%) | 5 (3.7%) | 1 (2.0%) | 4 (3.3%) | 1 (5.3%) | 0.914 | 0.964 |
| Copilot (Microsoft) | 25 (7.7%) | 8 (6.0%) | 8 (16.3%) | 5 (4.1%) | 4 (21.1%) | **0.006** | 0.527 |
| DeepSeek (High-Flyer) | 26 (8.0%) | 10 (7.5%) | 5 (10.2%) | 9 (7.4%) | 2 (10.5%) | 0.899 | 0.841 |
| Gemini (Google) | 56 (17.3%) | 27 (20.1%) | 6 (12.2%) | 16 (13.2%) | 7 (36.8%) | **0.044** | 0.846 |
| Grok (xAI) | 10 (3.1%) | 3 (2.2%) | 1 (2.0%) | 4 (3.3%) | 2 (10.5%) | 0.259 | 0.179 |
| Perplexity | 36 (11.1%) | 16 (11.9%) | 3 (6.1%) | 14 (11.6%) | 3 (15.8%) | 0.622 | 0.832 |
| Other | 42 (13.0%) | 25 (18.7%) | 4 (8.2%) | 12 (9.9%) | 1 (5.3%) | 0.078 | **0.020** |
| None | 41 (12.7%) | 11 (8.2%) | 9 (18.4%) | 18 (14.9%) | 3 (15.8%) | 0.209 | 0.104 |
| **Chatbot application areas** |  |  |  |  |  |  |  |
| Preparation of medical texts (e.g. doctor's letters, surgical reports) | 144 (44.6%) | 68 (50.7%) | 24 (49.0%) | 46 (38.0%) | 6 (31.6%) | 0.119 | **0.019** |
| Scientific writing (e.g. publications, study protocols, ethics applications) | 166 (51.4%) | 74 (55.2%) | 30 (61.2%) | 56 (46.3%) | 6 (31.6%) | 0.074 | **0.038** |
| Research (e.g. data analysis) | 114 (35.3%) | 55 (41.0%) | 17 (34.7%) | 34 (28.1%) | 8 (42.1%) | 0.166 | 0.111 |
| Teaching and training (e.g. case studies, presentations, examination questions) | 100 (31.0%) | 47 (35.1%) | 18 (36.7%) | 28 (23.1%) | 7 (36.8%) | 0.133 | 0.129 |
| Patient communication (e.g. texts written in layman's terms, surgical information) | 31 (9.6%) | 13 (9.7%) | 5 (10.2%) | 11 (9.1%) | 2 (10.5%) | 0.994 | 0.940 |
| Preparation and structuring of SOPs, checklists or treatment algorithms | 64 (19.8%) | 26 (19.4%) | 5 (10.2%) | 28 (23.1%) | 5 (26.3%) | 0.240 | 0.330 |
| Translation of medical texts (e.g. for international patients) | 124 (38.4%) | 58 (43.3%) | 16 (32.7%) | 42 (34.7%) | 8 (42.1%) | 0.417 | 0.277 |
| Other | 16 (5.0%) | 4 (3.0%) | 2 (4.1%) | 8 (6.6%) | 2 (10.5%) | 0.370 | 0.086 |
| None | 49 (15.2%) | 15 (11.2%) | 8 (16.3%) | 21 (17.4%) | 5 (26.3%) | 0.264 | 0.061 |
| ^1^n (%)  ^2^Pearson's Chi-squared test; NA  ^3^Chi-square trend test | | | | | | | |
|  | | | | | | | |
