# Supplementary material for: Artificial intelligence and chatbots in general surgery: a survey among surgeons in Germany, Austria and Switzerland
Source: Surg Endosc. 2026 Jul 1;40(9):7473–83. doi: 10.1007/s00464-026-13044-5 (PMC13582102; doi:10.1007/s00464-026-13044-5)
Supplement: Supplementary file 5 [file 464_2026_13044_MOESM5_ESM.docx]

**Supplementary Table 5** Perceived barriers and requirements for the routine use of AI in surgery

| **Characteristic** | **Overall**  N = 323^1^ | **Resident physician** n=134 (41.5%)^1^ | **Consultant** n=49 (15.2%)^1^ | **Senior physician** n=121 (37.5%)^1^ | **Head physician** n=19 (5.9%)^1^ | **p-value**^2^ | **p-value**^3^ |
| --- | --- | --- | --- | --- | --- | --- | --- |
| **Perceived barriers to the use of AI** |  |  |  |  |  |  |  |
| Lack of validated applications | 191 (59.1%) | 81 (60.4%) | 33 (67.3%) | 65 (53.7%) | 12 (63.2%) | 0.383 | 0.447 |
| Lack of high-quality annotated data sets for AI training | 142 (44.0%) | 67 (50.0%) | 18 (36.7%) | 49 (40.5%) | 8 (42.1%) | 0.303 | 0.149 |
| Lack of integration into existing systems | 249 (77.1%) | 104 (77.6%) | 38 (77.6%) | 91 (75.2%) | 16 (84.2%) | 0.846 | 0.971 |
| Data protection and legal uncertainties | 213 (65.9%) | 90 (67.2%) | 28 (57.1%) | 85 (70.2%) | 10 (52.6%) | 0.233 | 0.863 |
| Ethical aspects | 77 (23.8%) | 30 (22.4%) | 8 (16.3%) | 35 (28.9%) | 4 (21.1%) | 0.323 | 0.362 |
| Scepticism / lack of acceptance in the team | 103 (31.9%) | 42 (31.3%) | 19 (38.8%) | 41 (33.9%) | 1 (5.3%) | 0.057 | 0.410 |
| High demand for training | 85 (26.3%) | 39 (29.1%) | 11 (22.4%) | 30 (24.8%) | 5 (26.3%) | 0.787 | 0.485 |
| Lack of acceptance by patients | 29 (9.0%) | 14 (10.4%) | 5 (10.2%) | 9 (7.4%) | 1 (5.3%) | 0.773 | 0.312 |
| Lack of ability to empathise | 45 (13.9%) | 20 (14.9%) | 5 (10.2%) | 16 (13.2%) | 4 (21.1%) | 0.675 | 0.934 |
| Reduction of medical staff | 26 (8.0%) | 10 (7.5%) | 3 (6.1%) | 11 (9.1%) | 2 (10.5%) | 0.887 | 0.545 |
| Additional costs | 97 (30.0%) | 34 (25.4%) | 15 (30.6%) | 36 (29.8%) | 12 (63.2%) | **0.010** | **0.026** |
| Lack of approval for medical use | 152 (47.1%) | 70 (52.2%) | 23 (46.9%) | 50 (41.3%) | 9 (47.4%) | 0.385 | 0.128 |
| Unpredictable influence of technology companies | 112 (34.7%) | 42 (31.3%) | 15 (30.6%) | 46 (38.0%) | 9 (47.4%) | 0.398 | 0.123 |
| Reduction of skills of subsequent generations of surgeons | 96 (29.7%) | 31 (23.1%) | 17 (34.7%) | 40 (33.1%) | 8 (42.1%) | 0.145 | **0.034** |
| Other | 11 (3.4%) | 4 (3.0%) | 4 (8.2%) | 3 (2.5%) | 0 (0.0%) | 0.219 | 0.574 |
| None | 0 (0.0%) | 0 (0.0%) | 0 (0.0%) | 0 (0.0%) | 0 (0.0%) |  |  |
| **Requirements for routine use of AI** |  |  |  |  |  |  |  |
| Reliability of systems | 246 (76.2%) | 105 (78.4%) | 38 (77.6%) | 87 (71.9%) | 16 (84.2%) | 0.513 | 0.514 |
| Transparency in decision-making (explainability) | 189 (58.5%) | 81 (60.4%) | 29 (59.2%) | 66 (54.5%) | 13 (68.4%) | 0.622 | 0.685 |
| Increased AI training/education | 136 (42.1%) | 57 (42.5%) | 25 (51.0%) | 48 (39.7%) | 6 (31.6%) | 0.429 | 0.401 |
| Clear legal framework | 233 (72.1%) | 93 (69.4%) | 38 (77.6%) | 88 (72.7%) | 14 (73.7%) | 0.739 | 0.541 |
| Positive experiences of colleagues | 57 (17.6%) | 23 (17.2%) | 8 (16.3%) | 23 (19.0%) | 3 (15.8%) | 0.964 | 0.818 |
| Improved technical infrastructure of the clinic | 221 (68.4%) | 104 (77.6%) | 33 (67.3%) | 73 (60.3%) | 11 (57.9%) | **0.019** | **0.002** |
| Support from superiors/clinic management | 132 (40.9%) | 68 (50.7%) | 19 (38.8%) | 41 (33.9%) | 4 (21.1%) | **0.012** | **0.001** |
| Other | 8 (2.5%) | 3 (2.2%) | 1 (2.0%) | 3 (2.5%) | 1 (5.3%) | 0.878 | 0.624 |
| None | 3 (0.9%) | 1 (0.7%) | 0 (0.0%) | 2 (1.7%) | 0 (0.0%) | 0.711 | 0.659 |
| ^1^n (%) | | | | | | | |
| ^2^Pearson's Chi-squared test; NA | | | | | | | |
| ^3^Chi-square trend test | | | | | | | |
