# Supplementary material for: Artificial intelligence and chatbots in general surgery: a survey among surgeons in Germany, Austria and Switzerland
Source: Surg Endosc. 2026 Jul 1;40(9):7473–83. doi: 10.1007/s00464-026-13044-5 (PMC13582102; doi:10.1007/s00464-026-13044-5)
Supplement: Supplementary file 6 [file 464_2026_13044_MOESM6_ESM.docx]

**Supplementary Table 6** Potential of AI in surgical practice – assessment of improvement in surgical care quality through AI, need for AI training and 5-year outlook

| **Characteristic** | **Overall**  N = 323^1^ | **Resident physician** n=134 (41.5%)^1^ | **Consultant** n=49 (15.2%)^1^ | **Senior physician** n=121 (37.5%)^1^ | **Head physician** n=19 (5.9%)^1^ | **p-value**^2^ | **p-value**^3^ |
| --- | --- | --- | --- | --- | --- | --- | --- |
| **Quality improvement of surgical care through AI** |  |  |  |  |  | 0.451 | 0.331 |
| Disagree | 1 (0.3%) | 0 (0.0%) | 0 (0.0%) | 1 (0.8%) | 0 (0.0%) |  |  |
| Somewhat disagree | 9 (2.8%) | 2 (1.5%) | 3 (6.1%) | 3 (2.5%) | 1 (5.3%) |  |  |
| Neutral | 44 (13.6%) | 16 (11.9%) | 9 (18.4%) | 18 (14.9%) | 1 (5.3%) |  |  |
| Somewhat agree | 163 (50.5%) | 71 (53.0%) | 21 (42.9%) | 63 (52.1%) | 8 (42.1%) |  |  |
| Strongly agree | 103 (31.9%) | 45 (33.6%) | 15 (30.6%) | 35 (28.9%) | 8 (42.1%) |  |  |
| [NA] Not answered | 3 (0.9%) | 0 (0.0%) | 1 (2.0%) | 1 (0.8%) | 1 (5.3%) |  |  |
| **Need for AI training** |  |  |  |  |  | 0.349 | 0.206 |
| Disagree | 1 (0.3%) | 0 (0.0%) | 0 (0.0%) | 1 (0.8%) | 0 (0.0%) |  |  |
| Somewhat disagree | 5 (1.5%) | 1 (0.7%) | 2 (4.1%) | 2 (1.7%) | 0 (0.0%) |  |  |
| Neutral | 40 (12.4%) | 15 (11.2%) | 6 (12.2%) | 16 (13.2%) | 3 (15.8%) |  |  |
| Somewhat agree | 157 (48.6%) | 64 (47.8%) | 24 (49.0%) | 57 (47.1%) | 12 (63.2%) |  |  |
| Strongly agree | 118 (36.5%) | 54 (40.3%) | 17 (34.7%) | 44 (36.4%) | 3 (15.8%) |  |  |
| [NA] Not answered | 2 (0.6%) | 0 (0.0%) | 0 (0.0%) | 1 (0.8%) | 1 (5.3%) |  |  |
| **Use of AI in the next 5 years** |  |  |  |  |  | 0.698 | 0.226 |
| Very critical | 1 (0.3%) | 0 (0.0%) | 0 (0.0%) | 1 (0.8%) | 0 (0.0%) |  |  |
| Somewhat critical | 10 (3.1%) | 3 (2.2%) | 1 (2.0%) | 5 (4.1%) | 1 (5.3%) |  |  |
| Neutral | 28 (8.7%) | 8 (6.0%) | 6 (12.2%) | 14 (11.6%) | 0 (0.0%) |  |  |
| Somewhat positive | 184 (57.0%) | 80 (59.7%) | 27 (55.1%) | 67 (55.4%) | 10 (52.6%) |  |  |
| Very positive | 96 (29.7%) | 42 (31.3%) | 14 (28.6%) | 33 (27.3%) | 7 (36.8%) |  |  |
| [NA] Not answered | 4 (1.2%) | 1 (0.7%) | 1 (2.0%) | 1 (0.8%) | 1 (5.3%) |  |  |
| **Potential of AI in surgical practice** |  |  |  |  |  |  |  |
| Preoperative phase (e.g. diagnostics, therapy decision, surgical planning, prognosis) | 182 (56.3%) | 67 (50.0%) | 31 (63.3%) | 72 (59.5%) | 12 (63.2%) | 0.262 | 0.097 |
| Intraoperative phase (e.g. robotics, navigation, image guidance, real-time analysis, perfusion status) | 115 (35.6%) | 40 (29.9%) | 14 (28.6%) | 52 (43.0%) | 9 (47.4%) | 0.072 | **0.015** |
| Postoperative phase (e.g. complication detection, progression prognoses, automated documentation) | 151 (46.7%) | 71 (53.0%) | 23 (46.9%) | 48 (39.7%) | 9 (47.4%) | 0.209 | 0.065 |
| Training and simulation | 155 (48.0%) | 63 (47.0%) | 25 (51.0%) | 56 (46.3%) | 11 (57.9%) | 0.772 | 0.740 |
| Research (e.g. literature analysis, evidence mining, data analysis) | 226 (70.0%) | 96 (71.6%) | 38 (77.6%) | 80 (66.1%) | 12 (63.2%) | 0.425 | 0.254 |
| Quality assurance (e.g. big data analyses) | 193 (59.8%) | 77 (57.5%) | 27 (55.1%) | 76 (62.8%) | 13 (68.4%) | 0.616 | 0.258 |
| Other | 7 (2.2%) | 6 (4.5%) | 0 (0.0%) | 1 (0.8%) | 0 (0.0%) | 0.116 | **0.036** |
| None | 1 (0.3%) | 0 (0.0%) | 0 (0.0%) | 1 (0.8%) | 0 (0.0%) | 0.643 | 0.360 |
| ^1^n (%) | | | | | | | |
| ^2^Pearson's Chi-squared test | | | | | | | |
| ^3^Chi-square trend test | | | | | | | |
